# Host response transcriptional profiling reveals extracellular components and ABC (ATP-binding cassette) transporters gene enrichment in typhoid fever-infected Nigerian children

**DOI:** 10.1186/1471-2334-11-241

**Published:** 2011-09-13

**Authors:** Sok Kean Khoo, David Petillo, Mrutyunjaya Parida, Aik Choon Tan, James H Resau, Stephen K Obaro

**Affiliations:** 1 Laboratory of Microarray Technology, Van Andel Research Institute, Grand Rapids, MI, USA; 2 Laboratory of Cancer Genetics, Van Andel Research Institute, Grand Rapids, MI, USA; 3 Department of Medicine, University of Colorado Denver, Aurora, CO, USA; 4 Laboratory of Analytical, Cellular, and Molecular Microscopy, Van Andel Research Institute, Grand Rapids, MI, USA; 5 Department of Pediatrics and Human Development, College of Human Medicine, Michigan State University, East Lansing, MI, USA

## Abstract

**Background:**

*Salmonella enterica *serovar Typhi (*S*. Typhi) is a human-specific pathogen that causes typhoid fever, and remains a global health problem especially in developing countries. Its pathogenesis is complex and host response is poorly understood. In Africa, typhoid fever can be a major cause of morbidity in young infected children. The onset of the illness is insidious and clinical diagnosis is often unreliable. Gold standard blood culture diagnostic services are limited, thus rapid, sensitive, and affordable diagnostic test is essential in poor-resourced clinical settings. Routine typhoid fever vaccination is highly recommended but currently licensed vaccines provide only 55-75% protection. Recent epidemiological studies also show the rapid emergence of multi-drug resistant *S*. Typhi strains. High-throughput molecular technologies, such as microarrays, can dissect the molecular mechanisms of host responses which are *S*. Typhi-specific to provide a comprehensive genomic component of immunological responses and suggest new insights for diagnosis and treatment.

**Methods:**

Global transcriptional profiles of *S*. Typhi-infected young Nigerian children were obtained from their peripheral blood and compared with that of other bacteremic infections using Agilent gene expression microarrays. The host-response profiles of the same patients in acute vs. convalescent phases were also determined. The top 96-100 differentially-expressed genes were identified and four genes were validated by quantitative real-time PCR. Gene clusters were obtained and functional pathways were predicted by DAVID (Database for Annotation, Visualization and Integrated Discovery).

**Results:**

Transcriptional profiles from *S*. Typhi-infected children could be distinguished from those of other bacteremic infections. Enriched gene clusters included genes associated with extracellular peptides/components such as lipocalin (*LCN2*) and systemic immune response which is atypical in bacterial invasion. Distinct gene expression profiles can also be obtained from acute vs. convalescent phase during typhoid fever infection. We found novel down-regulation of ABC (ATP-binding cassette) transporters genes such as *ABCA7*, *ABCC5*, and ABCD4 and ATPase activity as the highest enriched pathway.

**Conclusions:**

We identified unique extracellular components and ABC transporters gene enrichments in typhoid fever-infected Nigerian children, which have never been reported. These enriched gene clusters may represent novel targeted pathways to improve diagnostic, prognostic, therapeutic and next-generation vaccine strategies for typhoid fever in Africa.

## Background

*Salmonella enterica *serovar Typhi (*S*. Typhi) is a Gram-negative bacterium that causes typhoid fever. The World Health Organization (WHO) recognizes *S*. Typhi infection as a global health problem, with an estimated 21 million cases and between 200,000 and 600,000 deaths annually [1,2]. In Africa, typhoid fever affects mainly school-age children and younger adults [3]. In fact, in endemic and large outbreak areas, young children represent a subgroup with the highest burden of infection as well as high rates of morbidity and complications [4]. The clinical diagnosis is often unreliable and confused with other febrile illness. Definitive diagnosis through blood or bone-marrow culture is laborious, expensive, and/or invasive, with sensitivity of only 40 to 70% [2,5]. Moreover, it takes several days for isolation and identification that can cause a delay to initiate proper antibiotic treatment. On the other hand, serologic diagnostic tests such as Widal tests lack sensitivity and specificity [6,7]. Lack of precision in diagnosis often implies non-targeted use of broad spectrum antibiotics which leads to the rise of multi-drug resistant strains [8]. A primary preventive strategy for disease prevention is vaccination. However, protective efficacies of both internationally licensed vaccines, Vi and Ty21, are modest (< 75%) [9,10]. As long as the understanding of pathogenesis of typhoid fever remains incomplete, improvement on diagnosis, treatment and vaccine strategies will be subsequently delayed.

One of the approaches to elucidate the pathogenesis of typhoid fever is to better understand the mechanisms of virulence and susceptibility of hosts. Host responses to bacterial infections are determined by specific structural and antigenic components of the bacteria and the profile of immunological response should be unique to different classes of bacteria or bacteria species. Global transcriptional profiling of the peripheral blood using microarrays has been proven feasible to identify distinct host responses in various diseases [11-16]. In the field of infectious diseases, a host's immune response to a certain pathogen can be clearly defined and its unique molecular signature identified using high-throughput microarray technology, eliminating the limitation of conventional diagnostic or microbiological techniques.

This is the first host response transcriptional profiling study on young children with typhoid fever in Africa. Our preliminary observations showed distinct transcriptional profiles between *S*. Typhi vs. other bacteremic-infected children, as well as acute vs. convalescent phase. We identified novel sets of genes associated with extracellular components which are specific to *S*. Typhi infection, as well as the ABC transporters gene cluster which can discriminate acute from convalescence phases.

## Methods

### Subject recruitment

This is a sub study for our CABSYNC (Community- Acquired Bacteremic Syndrome in Young Nigerian Children) surveillance program for children aged 2 months to 14 years in central Nigeria. Children with fever (temperature ≥ 38.5°C) and any of the following: respiratory distress, convulsion or diarrhea, were eligible for enrollment. Since the use of antibiotics could modify the transcriptional profile, only treatment-naïve patients were recruited. Blood was drawn using aseptic technique for culture into an aerobic Pediatric plus Bactec culture bottle (Becton Dickinson (BD)) which contains antibiotic resins. The bottles were incubated for no longer than 5 days in the Bactec 9050 instrumented blood culture systems (BD). Positive specimens were Gram stained and sub-cultured on appropriate agar plates. After primary isolation and characterization, secondary confirmation was performed at the Medical Research Council Laboratories, Gambia, or Sparrow Regional Laboratories, East Lansing, Michigan, USA. Salmonella isolates were further characterized at the Sanger Institute, Oxford, United Kingdom. Antibiotic susceptibility testing was performed using standard methods. Antimicrobial activity in serum was detected using the Micrococcus luteus assay. Blood from acute (before initiating antibiotics) and convalescent (4 weeks after acute presentation) phases of the same patients were sampled. Parental consents were obtained from all children in this study. This study was approved by the Institutional Review Board of Michigan State University and the National Hospital, and the Ethics committee of the Federal Capital Territory Abuja, Nigeria.

### RNA extraction and purification

Two to five milliliters of peripheral blood were drawn from each subject into a PAXgene blood RNA tube (BD). PAXgene tubes contain a proprietary reagent that immediately stabilizes intracellular RNA for 3 days at 18-25°C and 5 days at 2-8°C, and thereafter at -70°C for long-term storage. The PAXgene blood RNA system is a product approved by the US FDA for collection, storage, and transport of blood for molecular diagnostic testing. Extraction and purification protocols were performed according to PAXgene blood RNA kit (Qiagen). The quality and quantity of RNA was measured on a RNA nano microfluidic chip using the BioAnalyzer (Agilent Technologies).

### Microarray

Whole human genome 4 × 44K gene expression 2-color microarrays from Agilent were used in this study. In brief, 200 ng of total RNA was amplified, fluorescently labeled, and hybridized onto the arrays according to the manufacturer's protocols. RNA from acute phase was labeled with Cy3 (green dye) and convalescence phase with Cy5 (red dye). After hybridization, the arrays were washed and scanned with an Agilent scanner. Probe features were extracted from the microarray scan data using Feature Extraction software v.9.5.3.1 (Agilent). Microarray data were deposited in Gene Expression Omnibus (GEO) with the accession number #GSE28658.

### Gene expression analysis

The gene expression of each sample was loaded into the R environment and the Limma package was applied for analysis. Data were background corrected in order to obtain accurate intensity values while red and green channels were separated and quantile normalization was applied to remove systemic/technical variations before statistical analysis. A linear model was applied before eBayes test and top up- or down-regulated genes were generated. The statistic used for significance analysis was the moderated t-test. This has the same interpretation as a standard t-test except that the standard errors were moderated across genes, i.e., shrunk towards a common value, using a simple Bayesian model. As the number of samples is small, we chose to use the moderated t-test in this pilot study as an exploratory approach to identify differentially- expressed genes.

### Pathway analysis

The Database for Annotation, Visualization and Integrated Discovery (DAVID) Bioinformatics Resources v.6.7 from the National Institute of Allergy and Infectious Diseases (NIAID/NIH) http://david.abcc.ncifcrf.gov/, a gene-centered database integrating heterogeneous gene annotation resources, was applied to facilitate high-throughput gene functional/pathway analysis [17,18]. The group enrichment score, the geometric mean (in -log scale) of member's *P*-values in a corresponding annotation cluster, is used to rank their functional significance.

### Quantitative Real time PCR (Q-PCR)

To validate the microarray results, four genes with differential expression (*MYPOP*, *PSAP*, *IL11RA*, and *MUC20*) were evaluated using Q-PCR (Table 1). All RNA samples were diluted to the same concentration (500 ng/μl), and the same quantity, 5 μl, (2.5 μg total RNA) was used for reverse transcription to cDNA, according to standard ABI protocols, using the TaqMan PCR (Applied Biosystems (ABI)). cDNA samples were then quantitated with a NanoDrop spectrophotometer (Thermo-Scientific), using the same total cDNA quantities for each sample for Q-PCR assay on an ABI StepOne Plus instrument (ABI). Single-plex PCR reactions were carried out in triplicate using TaqMan Universal PCR Master Mix with no UNG (ABI). Q-PCR data were initially collected and a preliminary analysis was performed using the StepOne Software version 2.1. Controls included human beta-actin (endogenous gene reference), an ABI Control Total RNA (human positive control) and nuclease-free water (non-template negative control). Further Q-PCR analyses were performed with the comparative 2-ΔΔC_T _quantitation method [19].

**Table 1:** Specific TaqMan gene expression assays for Q-PCR and amplicon lengths

Gene	TaqMan assay ID	Amplicon length (bp)
*MYPOP*	Hs02384801_m1	72
*PSAP*	Hs01551096_m1	74
*IL11RA*	Hs00234415_m1	120
*MUC20*	Hs00416321_m1	83

## Results and discussion

### Clinical data of patients and feasibility of recruitment

During this pilot study, 12 patients were recruited. Six patients were excluded for the microarray study due to unavailability of convalescent phase sample, no bacteria growth from blood cultures, or insufficient or low quality RNA. Three patients with *S*. Typhi infection and one each of Acinetobacter, Klebsiella, and non-typhoidal salmonella infection of acute and convalescent phases were profiled using gene expression microarrays. The clinical characteristics of the patients are presented in Table 2.

**Table 2:** Clinical data of Nigerian children with *S*. Typhi and other bacteremic infections

Sample	Age	Sex	Bacteria Type	Clinical Symptoms
01	6 yrs 4 mo	F	*S*. Typhi	fever 14 days, diarrhea 4 days
03	3 yrs 4 mo	F	Acinetobacter spp	fever 14 days, cough with difficult breathing 1 day
05	9 mo	F	non-typhodal Salmonella	fever 5 days
08	6 yrs 4 mo	M	Klebsiella spp	fever, vomiting, diarrhea 5 days, cough 7 days
09	5 yrs 1 mo	M	*S*. Typhi	fever 30 days, diarrhea 14 days
11	12 yrs 1 mo	F	*S*. Typhi	fever 30 days, diarrhea 14 days

Previously, we showed that typhoid fever is the most prominent infectious disease among Nigerian children from our CABSYNC (Community- Acquired Bacteremic Syndrome in Young Nigerian Children) surveillance data between September 2008 and November 2009, screening 1,287 children aged 2 months to 5 years [20]. Therefore, it is critical to elucidate the molecular mechanisms of host response in African children with typhoid fever, an under-represented yet extremely important research cohort. Transcriptional microarray studies require intracellular RNA which can be a challenge since RNA can be unstable and rapidly degrade within hours after blood collection. In this pilot study, we showed that it is feasible to collect whole blood samples from African children using PAXgene Blood RNA tubes which contain an additive that stabilizes the *in vivo *gene transcription profile by reducing *in vitro *RNA degradation and minimizing gene induction. Moreover, PAXgene tubes can be safely stored or transported at 15-25°C for up to 3 days or at 2-8°C for up to 5 days, thus suitable for blood collection in freezer-lacking rural areas in Africa.

### Transcriptional profiles of *S*. typhi patients

**Figure 1:**
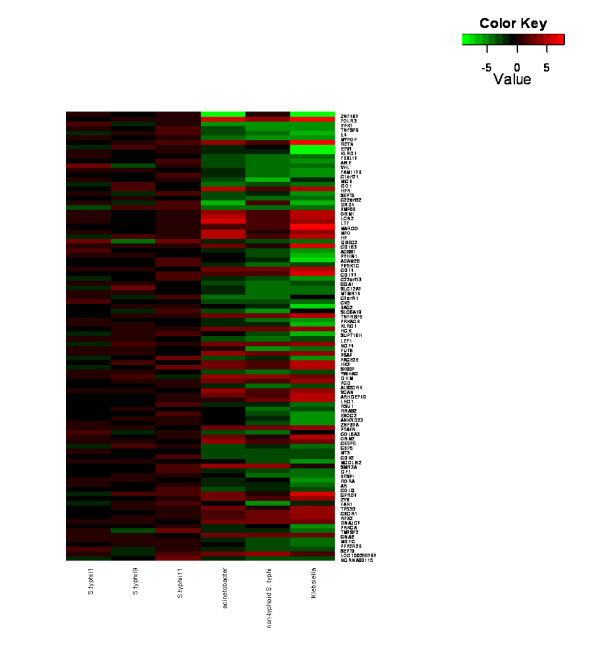
**Distinct transcriptional profiles of *S*. Typhi patients**. A heatmap of differentially-expressed genes showing distinct transcriptional profiles of *S*. Typhi patients compared with patients with Acinetobacter, non-typhoidal salmonella or Klebsiella infection. Red indicates over-expressed transcripts and green represents under-expressed transcripts.

**Figure 2:**
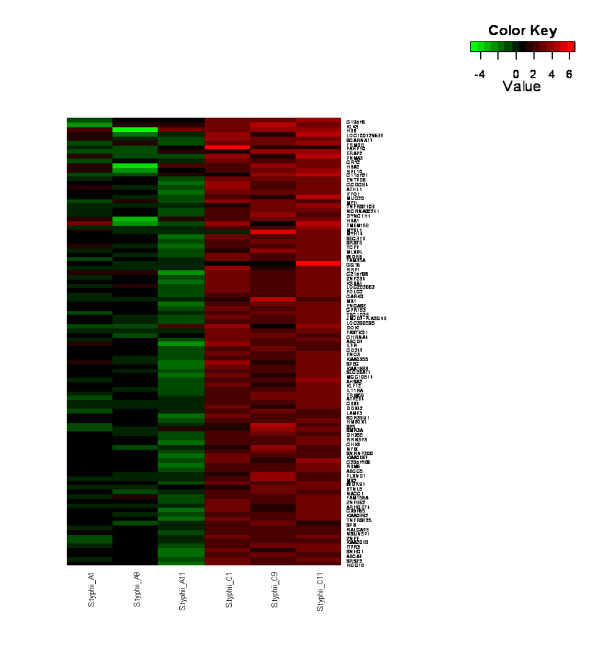
**Distinct transcription profiles of acute vs. convalescence phase**. A heatmap of differentially-expressed genes showing distinct profiles between acute (A) and convalescence (C) phase of *S*. Typhi infection. Red indicates over-expressed transcripts and green represents under-expressed transcripts.

To determine whether global transcriptional profiles of *S*. Typhi patients were distinct from those of other bacteremic infections, namely Acinetobacter, Klebsiella, and non-typhoidal salmonella, the average signals after normalization and background correction of each group during acute phase were compared using the moderate t-test statistic. Using this exploratory statistical approach, we focused on up to 100 top up- and down-regulated genes with *P *< 0.05 and fold change > 2.5. A heatmap of top up- and down-regulated genes was generated, revealing distinctive transcriptional profiles of *S*. Typhi patients in comparison with other bacteremic infections (Figure 1). To determine the host response transcriptional profiles at acute vs. convalescence phase, gene expressions during these phases were compared in the same typhoid fever patients. Our results showed that the patterns of transcriptional profiles at acute phase were distinctive from the convalescence phase in *S*. Typhi-infected children (Figure 2).

### *S*. Typhi infection-specific genes and pathways

The top 96 differentially-expressed genes (*P *< 0.05, fold change > 2.5) between *S*. Typhi-infected children and other bacteremia (Figure 1)(Additional file 1) included genes that are involved in ligand binding and alteration of cell composition such as lipocalin (*LCN2*) and prosaposin (*PSAP*). *LCN2 *was down-regulated, which is an atypical response for bacterial invasion. In addition, genes associated with systemic inflammatory response such as interleukin 4 (*IL4*) and tumor necrosis factor (ligand) superfamily, member 9 (*TNFSF9*) were found up-regulated, which is another example of an atypical bacterial invasion response. We used DAVID to assign annotation groups based on gene ontologies and, as expected, top ranked clusters included genes related to extracellular proteins and components as well as systemic defense and inflammatory response pathways (Additional file 2).

In the human host, detection of pathogens is achieved through pattern-recognition receptors (PPRs) such as the membrane-localized Toll-like receptors (TLRs) [21] and the cytosolic nucleotide-binding and oligomerization domain-like receptors [22] of the host innate immune-surveillance system. PRRs function as bar-code readers that recognize conserved molecular structures in microbes known as microbe-associated molecular patterns [23]. With a focus on the extracellular protein and component gene clusters (highest enrichment score in our study), we found down-regulation (-3.6 fold) of *LCN2 *(lipocalin-2 or neutrophil gelatinase-associated lipocalin) in typhoid fever children intriguing. Lipocalins are extracellular proteins that display different molecular recognition properties such as ligand and receptor binding. Hence, they are known to bind with small hydrophobic molecules, cell surface receptors, as well as form complexes with other macromolecules. The invariant end of the lipocalin fold may implicate the general binding to common cell surface receptors, while the more variable end is adapted to specialized ligand binding and forming of macromolecular complexes [24]. During bacterial invasion, TLRs in immune cells stimulates transcription, translation, and secretion of lipocalin 2 protein (Lcn2) to sequester the siderophore enterobactin (Ent) and inhibit intracellular microbial growth by blocking iron intake [25]. Lcn2 may also represent a novel mechanism of sensing microbial metabolism to modulate the host response [26]. Lcn2 can be up-regulated by pro-inflammatory cytokines such as IL-17, IL-22, and IL-1α to provide a robust innate response in a typical microbial infection [27-30]. However, we found *LCN2 *down-regulated in typhoid fever children, an observation of an atypical antibacterial response. One explanation is that having the *iroA *gene cluster in some bacteria such as Salmonella spp allows bacteria to evade this effector mechanism of the innate immune system, rejuvenating their Ent-mediated iron-acquisition pathway and play a role in their virulence [31]. One can also debate the potential of the hydrophobic adhesion protein of *S *Typhi such as T2544 [32] which binds directly to Lcn2 or TLRs which bind to Lcn2 and causes transcriptional disruption and down-regulation of *LCN2*. If so, such hydrophobic adhesion proteins can be a potential immune-modulatory target for typhoid fever. In general, our findings on differentially-expressed genes in typhoid fever children can lead us to explore the genes related with extracellular proteins and/or atypical bacterial inflammatory response for future development of typhoid fever-specific diagnostic tools/biomarker panels and anti-typhoid fever strategies and therapies.

It has been suggested that host responses are often typical for groups of pathogens instead of being specific to individual pathogens, as some pathogens share basic clinical characteristics such as fever and diarrhea [33]. However, *S*. Typhi is known to be an atypical bacterium which does not elicit a typical antibacterial host response characterized by exudative intestinal inflammation, but a nonspecific response of interstitial inflammation resembling viral or parasitic infections [33]. Here, we showed up-regulation of *IL4 *and *TNFSF9 *in *S*. Typhi patients. An increased *IL4 *level is known to be associated with immune system response in chronic viral hepatitis [34] while *TNFSF9 *is reported over-expressed in primary biliary cirrhosis, an autoimmune disease of medium and small bile ducts [35]. Both are cytokines associated with T-cells which have protective capability for systemic inflammation. We also showed down-regulation of *HRP *(haptoglobin-related protein) and *HP *(haptoglobin), indicating a systemic response. *HPR *is commonly regulated as the innate host immunity against Trypanosoma, a protozoan parasite [36,37]. Our results reinforced the opinion that *S*. Typhi does not elicit classic antibacterial host responses.

### Expressed genes and pathways during acute and convalescence phases

The distinct transcriptional profile of the acute phase consisted of genes related with hemoglobin (hemogloblin-beta, *HBB*; hemoglobin subunit alpha 1 and 2, *HBA 1 and HBA2*) and inflammatory (interleukin 11 receptor, alpha, *IL11RA*; mucin 20, cell surface associated, *MUC20*) that were down-regulated but up-regulated in the convalescence phase (Figure 2)(Additional file 3). Interestingly, down-regulation of ABC transporter genes (ATP-binding cassette, subfamily A, member 7, *ABCA7*; ATP-binding cassette, subfamily C, member 5, *ABCC5*; ATP-binding cassette, subfamily D, member 4, *ABCD4*) and enrichment of this pathway in the acute phase of typhoid fever patients were detected and the ATPase activity molecular function was clustered as a top-scoring biological function (Additional file 4).

Genes associated with the distinct transcript profiles at different phases of illness have been identified in a cohort of Vietnamese typhoid fever patients (age unknown) [38]. Here, we presented transcriptional profiles and differentially-expressed genes of acute and convalescent phases in a small, unique cohort (young children with African descent) which represents the most prominent typhoid fever-infected yet under-represented group in Nigeria. Genes related to hemoglobin such as *HBB*, *HBA 1 *and *HBA2 *were down-regulated, reflecting the anemic characteristic of typhoid fever patients and are consistent with those reported in the Vietnamese cohort [38]. However, we found down-regulation of inflammatory-related genes such as *IL11RA *and *MUC20 *at the initial acute phase but up-regulated 4 weeks later in the convalescent phase. These results are different from the Vietnamese cohort where most immune response genes were over-expressed in acute phase and down-regulated later in convalescent phase. While we are uncertain whether the Vietnamese cohort was sampled before or after initiation of antibiotics (besides unknown age), our study involved young children (average age = 5 years 7 months) that were sampled before antibiotics prescription in their acute phase and 2 patients (09 and 11) have prolonged fever of 30 days. Thus, we can surmise that the innate response of our patients may not have been fully effective during the initial acute phase and took a longer time to recover (hence still maintaining up-regulation of immunological-related genes after 4 weeks) due to their young age and not fully developed/weaker immunity. In addition, it has been reported that a switch from pro-inflammatory to an anti-inflammatory mode occurred and inhibited production of cytokines in the acute phase of typhoid fever patients [39]. It can also be explained by the 'stealth' tactics of *S*. Typhi, evading recognition of TLR4 and TLR5, impairing identification of its invasion to prevent a typical antibacterial host response, which results in suppression of neutrophil recruitment [40] and weak induction of acute phase responses [41].

From our unique cohort, we also identified down-regulation of ABC transporter genes (ATP-binding cassette, subfamily A, member 7, *ABCA7*; ATP-binding cassette, subfamily C, member 5, *ABCC5*; ATP-binding cassette, subfamily D, member 4, *ABCD4*) and enrichment of this pathway in the acute phase of typhoid fever, which have never been previously reported. ABC proteins transport a wide variety of substrates ranging from small ions to macromolecules across the cell membrane. Its export systems in all living organisms are involved in the extrusion of harmful substances and export of extracellular toxins, which can be *S*. Typhi bacterium in our case, but needs further validation. The same explanation can be given that the ABC transporter genes were down-regulated in the initial acute phase but up-regulated 4 weeks later due to a slower and less efficient host response caused by young age and weaker immunity.

Human ATP-binding cassette transporter superfamily is also known to be involved in drug response phenotypes. Antimicrobial drug resistance of *S*. Typhi is well known [8] and our findings may shed light to the appropriate antibiotic treatment to prevent the development of resistant strains of S. Typhi which is an emerging problem. While the definite functions of *ABCA7 *and *ABCD4 *are unknown, *ABCC5 *is a member of the MRP (multi-resistance protein) subfamily and may contribute to resistance of certain anti-cancer and anti-HIV drugs [42-44]. Thus, whether *ABCC5 *plays a direct or indirect role in multi-drug resistance to typhoid fever antibiotics warrants further investigation.

Transcriptional profiles from the recovery phase were not available as these samples were not acquired during this pilot study. One would predict that most recovery profiles would fail to show distinctive signatures of acute and convalescent stages. However, patients who retain the convalescent signature in the recovery phase may be an indication of carrier state, relapse or susceptible to reinfection [38]. We are currently planning to include the recovery phase, beside acute and convalescent phases, in a follow-up study with a larger cohort (> 100 African children).

### Validation of microarray gene expression with Q-PCR

We confirmed the microarray data by Q-PCR that targeted four differentially-expressed genes, *MYPOP*, *PSAP*, *IL11RA*, and *MUC20*. These genes were selected based on their functions related to extracellular components or host responses. In our microarray data, *MYPOP *was up-regulated in *S*. typhi-infected patients but down-regulated in other bacteremic infections. Conversely, *PSAP *was down-regulated in *S*. typhi-infected patients but up-regulated in other bacteremic infections. Both *IL11RA *and *MUC20 *were down-regulated in acute but up-regulated in convalescent phase. In addition to the original samples used for microarray analysis, three new samples (two typhoid fever and one enterobacter) were obtained for Q-PCR validation. We demonstrated that the Q-PCR results of *MYPOP*, *PSAP*, *IL11RA*, and *MUC20 *were consistently correlated with the results observed in the initial microarray data (Table 3).

**Table 3:** Validation of gene expression using Q-PCR

	**Average fold change using **2-ΔΔC_T _**method**			
	***MYPOP***	***PSAP***	***IL11RA***	***MUC20***

***S*. Typhi infection**	2.9	1.6	-	-
**Other bacteremic infection**	1.7	2.7	-	-
***S*. Typhi acute phase**	-	-	1.8	1.9
***S*. Typhi convalescent phase**	-	-	3.2	4.1

## Conclusions

It is well known that current diagnostic tests for typhoid fever are inadequate in many ways and development of reliable and affordable diagnostic assays is critical to fill this gap for better disease control and treatment [45]. In this pilot study, we explored the global transcriptomes of Nigerian children with typhoid fever. This led to the characterization of distinct transcriptional profiles for children specifically with typhoid fever and during acute vs. convalescent phases. We showed that evaluating naïve patients (before any antibiotic treatment) may obtain more accurate molecular signatures for diagnostic and progression of typhoid fever. This unique cohort (treatment-naïve, average age of 5 years 7 months) is also more suitable for strategizing novel vaccines as young children are the preferred age for immunization to induce effective and long-lasting immunity. We confirmed that *S*. Typhi elicits atypical antibacterial host response. We also identified novel extracellular component and ABC transporters as key gene clusters in these young patients which may lead to a more complete understanding of typhoid fever. These enriched gene clusters may represent novel targeted pathways to improve diagnostic, prognostic, therapeutic and next-generation vaccine strategies of typhoid fever in Africa.

## Competing interests

The authors declare that they have no competing interests.

## Authors' contributions

SKK contributed to the microarray experiment study design, performed the RNA extraction and microarray procedures, and wrote the manuscript. DP carried out the real-time PCR assays and analyses. MP and ACT provided expertise in gene expression microarray analysis. JHR participated in its design and supervised the study. SKO conceived the study, participated in its design and coordination, and contributed to writing the manuscript. All authors read and approved the final manuscript.

## Pre-publication history

The pre-publication history for this paper can be accessed here:

http://www.biomedcentral.com/1471-2334/11/241/prepub

## Supplementary Material

Additional file 1 **Most differentially expressed genes for typhoid fever vs. other bacteremic infections**. Log fold change and *P *value of top 96 *S*. Typhi infection-specific genes. Click here for file

Additional file 2 **Top pathway clusters for typhoid fever vs. other bacteremic infections**. Enrichment score and *P *value of top 10 *S*. Typhi infection-specific functional annotation clusters, including extracellular proteins/components and systemic defense/inflammatory response pathways. Click here for file

Additional file 3 **Most differentially expressed genes during acute vs. convalescent phase of typhoid fever**. Log fold change and *P *value of top 100 up- and down- regulated genes during acute vs. convalescent phase of typhoid fever. Click here for file

Additional file 4 **Top pathway clusters for acute vs. convalescent phase of typhoid fever**. Enrichment score and *P *value of top 10 functional annotation clusters during acute vs. convalescent phase of typhoid fever, with ATPase activity as the highest-ranking molecular function. Click here for file
